# Overview of the mental health system in Mozambique: addressing the treatment gap with a task-shifting strategy in primary care

**DOI:** 10.1186/s13033-015-0032-8

**Published:** 2016-01-04

**Authors:** Palmira Fortunato dos Santos, Milton L. Wainberg, José Miguel Caldas-de-Almeida, Benedetto Saraceno, Jair de Jesus Mari

**Affiliations:** Mental Health Department, Center for Applied Psychology and Psychometric Tests, Ministry of Health, Rua de Nachingwea No 486, Maputo, Mozambique; New York State Psychiatric Institute, Columbia University, New York, USA; Lisbon Institute of Global Mental Health, Universidade Nova de Lisboa, Lisbon, Portugal; Lisbon Institute of Global Mental Health, Lisbon, Portugal; Department of Psychiatry, Universidade Federal de São Paulo, São Paulo, Brazil

**Keywords:** Mental health systems, Low income countries, Task-shifting, Mozambique, Psychiatric technician, Primary care, Scaling-up services

## Abstract

**Background:**

Mozambique has gradually changed its action on mental health (MH) from an asylum-centric care with long-term hospitalization to an innovative approach to community and primary care.

**Objective:**

To collect essential information on Mozambique’s MH system for decision making, to improve quality of services delivered, update MH Strategy and Action Plan.

**Method:**

The study used the WHO-AIMS to assess MH systems including policy and legislation, organization of services, MH in primary care, human resources, public education and link with other sectors, monitoring and research. A comparative analysis was conducted to present the evolution of relevant data from 2010 to 2014.

**Results:**

There are two psychiatric hospitals in the country and beds in general hospitals. In the period, the number of beds in general hospitals remained stable (203), and the beds in psychiatric hospitals increased from 173 to 298. Mental health outpatient facilities have increased from 83 to 152. The number of psychiatrists (9 in 2010, and 10 in 2014) remained very low, with a significant increase in the number of psychologists (56–109) and occupational therapists (2–23). The number of Psychiatric Technicians has increased from 66 in 2010, to 241 in 2014. This increase allowed the mental health network to expand from 60 to 135 Districts, meaning an increase of coverage from 44 to 100 % of the country districts.

**Conclusion:**

The task-shifting strategy focused on services delivered in primary care by psychiatric technicians, mid-level professionals, allowed the expansion of mental health services for all the country districts and the reduction of treatment gap in Mozambique.

## Background

Mental, neurological and substance use disorders are leading causes of the global burden of disease and profoundly impact the social and economic well-being of individuals and communities [1]. Between 35.5–50.3 % of MNS cases in developing countries do not receive any treatment. This is significantly increased in low and middle-income countries where numbers go up to 76.3–85.4 % [2, 3]. Resources for mental health such as policies and infrastructures, mental health services (community-based, beds, essential medicines), community resources (NGOs, consumer and family associations, and social networks), human resources and funding are scarce in low and middle-income countries [4]. Among The different barriers for the development of mental health in low- and middle-income countries, human resources scarcity is a major concern. One well-established barrier to scaling-up of mental health services is the inadequate number of people who are trained to provide care [4, 5].

Mozambique is a country located in Southern Africa with a geographic area, of 801,590 km^2^. The country became independent from Portugal in 1975 and inherited Portuguese as the official language [15]. However, it is the native language of only 6 % of the population, 25 % of which living in the capital city Maputo. There are three major ethno-linguistic groups in the country mainly the *Xitsonga* in the south, the *CiSena* in the central zone and the *eMacua* in the north. The main religions in the country are for the Catholic, Islamic and other Christian and animist religions [16]. According to the World Bank indicators of 2015, Mozambique is considered a Low Income Country [17] and a Low Human Development Country, occupying the 178th place out of 187 countries in the Human Development Index of the United Nations [18]. As per the 2007 census, Mozambique had a population of 20,530,714 inhabitants: 46.9 % of the population is under 14 years of age, and 3.1 % is over 65 years of age. Approximately 70.2 % of the population lives in rural areas [16]. Life expectancy at birth is 51.8 years for women and 47.1 for men. And the overall literacy rate is 49.6 % [16]. Country population increased to 25.727.911 in 2014 as seen in National Statistics Institute recent data [19].

As of 2007 National Inventory of Health Infrastructures, the National Health Service (Public Sector) has 84 hospital general beds and 5.7 general doctors for every 100,000 inhabitants. In terms of primary care, there are 1224 health facilities (health posts and centers) with or without a physician [20]. The resources for health are distributed throughout the country in response to the decentralization strategy in the National Health Policy of 2007 although there is greater concentration of specialists in the capital.

Resources for the development of mental health services are very scarce [21–26]. The Mental Health National Program is also community based with most patients treated in community primary health facilities or outpatient units.

In order to cope with these barriers Mozambique started a process to integrate mental health in primary care, in general health care and into priority health care platforms. The WHO services organization pyramid for an optimal mix of services for mental health [6, 7] is being implemented using task-shift/task-sharing models.

Task shifting, which refers to the strategy of rational redistribution of tasks among health workforce teams, has become a popular method for addressing shortages of specialist health resources. When appropriate, highly qualified health workers share specific tasks with health workers having less training and fewer qualifications in order to make more efficient use of the available human resources [8].

Mozambique decided to train Psychiatric Technicians (PT), who are mid level health professionals, trained to deliver psychiatric care including prescription of psychotropic medicines, promoting self-care, integrating MH services in general hospitals and community mental health services delivery aiming to reduce the number of patients admitted in mental hospitals.

The psychiatric technicians are trained in psychiatry and mental health in a 2.930 h course provided in 30 months, corresponding to 59.8 % theoretical and practical lectures, 22.2 % traineeship (curricular practice) and 18 % integrated internship (last course semester). The course is lectured by psychiatrists, neurologists, psychologists and other clinicians and includes subjects such as anatomy and physiology, anthropology, psychiatric symptomatology, child and adolescent psychiatry, psychology, neurology, psychopathology, community mental health and psychopharmacology. The course is lectured in three provinces (in the north, center and south of the country) and is directed by the mental health department. Students are selected by admission tests that include biology, Portuguese, and psychometric and vocational tests [9].

As result of this training, we have a new cadre of professionals to whom the main tasks are shifted from specialists (psychiatrists and clinical psychologists) in most settings responding to evidence based data from different studies and WHO recommendations. Tasks can be shifted and shared with specialists supporting primary care providers and community health workers to routinely identify patients who need care (case finding); assess risk factors; educate patients about their illnesses, risk factors, and treatment; intervene with a combination of brief evidence based pharmacological and psychosocial treatments; teach self-management skills; monitor patients’ progress and adherence to treatment; and follow-up over the long term [7, 10–14].

Experience with task-shifting and/or task sharing shows that many of the required skills and tasks of care can be learned and delivered by a range of non-specialist health workers with appropriate training and supervision. Particular skills, such as case finding, support of treatment adherence and motivational coaching, follow-up tracking, patient education, and self-management support, turn out to be quite critical to providing effective care [13].

The main objective of this study was to collect essential information on the mental health system in Mozambique that could be the basis for making recommendations for improving the quality of services delivered to the population and to contribute to updating the Mozambique Mental Health Strategy and Action Plan. Specifically, the study aimed to provide information about the strengths and weaknesses of the existing system outlining a starting point for the implementation of reforms in mental health services in the country.

## Methods

The study was conducted to present the evolution of relevant data from 2010 to 2014 based on a comparative analysis gathered from (1) a 2010 survey from Mental Health Department using the WHO-AIMS, a set of questionnaires developed by the WHO to assess mental health systems including policy and legislation, organization of mental health services, mental health in primary care, human resources, public education and link with other sectors, monitoring and research; and (2) Mental Health Department 2014 data reports regarding services evaluation and activities of the year 2014. The two data source covered information from all the health facilities with mental health services in the country, the province health directorates in all the ten provinces including the capital, and the Mental Health Department at Ministry of Health.

For the WHO-AIMS, the data collection instruments were translated, back-translated and then validated and adapted to Mozambican context. After back-translation a comparison between the original English version and the back-translated version was made to check if there were significant differences that could alter the response to the questionnaires. After being approved, the translated questionnaires were applied in two peripheral health units in the city and province of Maputo and in Mavalane Hospital which is located in an urban area in the city center.

A team of mental health professionals (psychologists, psychiatrists and psychiatric technicians) was trained to collect the data from health care facilities records, interviews and observational evaluation. There were two training sessions (total 8 h) to administer the questionnaires. Three psychologists gave training to the data collection team of the Department of Mental Health. This team was composed of psychologists and psychiatrists and led the research groups in each province. These researchers did a 2-h training session with local colleagues who have integrated teams in each province (usually two or three—psychologists, psychiatrists and psychiatric technicians) for data collection in the districts. Data collection took place from June to August 2010 in all the ten provinces of the country. A team from Mental Health Department conducted data collection in all health facilities with mental health services and at the administrative health directorates in each province. Data were filtered and summarized in Microsoft excel program and then introduced to the *WHO*-*AIMS Excel Data Entry Program*.

Mental health Department compiles annually data reports from information gathered from all the health facilities with mental health services that are compiled by the province coordinators. Data from the ten provinces are sent to Mental Health Department for the Mental Health Annual Report. Information regarding diagnostic, patients, human resources, services organization, psychotropic drugs availability, prevention and promotion activities were collected using a parallel information system that has been used since 2006 and has been improved as result of the WHO-AIMS report. For this study, data from 2014 compiled in January 2015 was the main source of information [26].

## Results

### Mental health services

Mental Health Department in the Ministry of Health is the entity responsible for coordinating mental health services in the country. It is through the National Mental Health Program that the mental health services are implemented nationally.

Countrywide, in 2010 there were 92 health facilities providing mental health services representing two mental hospitals, three psychiatric wards in General Hospitals, 4 day treatment facilities and 83 outpatient facilities (see Table 1). The outpatient services alone treated 131 patients per 100,000 inhabitants.

**Table 1:** The distribution of mental health facilities and human resources for the period 2010-2014

Data source	Total number of facilities/beds/professionals		Rate per 100,000 population	
	AIMS 2010	MISAU 2014	AIMS 2010	MISAU 2014
Population	20.530.714	25.727.911		
Settings				
Mental health outpatient facilities	83	152	0.35	0.59
Day treatment facilities	4	9	0.02	0.03
Psychiatric beds in general hospitals	203	203	0.98	0.78
Psychiatric hospitals	2	2	0.09	0.09
Beds in psychiatric hospitals	173	298	0.84	1.15
Providers				
Psychiatrists	9	10	0.04	0,04
General practitioners	2	0	0.01	0,00
Nurses	44	44	0.21	0,17
Psychologists	56	109	0.27	0,42
Social workers	5	7	0.02	0,03
Occupational therapists	2	23	0.01	0,09
Psychiatric technicians	66	241	0.32	0,94

In 2014 the number of health facilities providing mental health services increased to 153 covering all the districts of the country using a task-shift strategy where mid level professionals trained as psychiatric technicians delivered mental health services in primary health care units. This expansion of mental health services was only feasible due to the training of these professionals.

There are still only 4 day-care centers and the rate of utilization of services is less than 1.0 per 100,000 inhabitants in 2010 and 2014 data sources.

There are 29 community-based inpatient service units but services coverage is still below the needs. Users are less than 1.0 per 100.000 inhabitants. The number of beds (203) in these facilities remains the same in 2014 following the goals of the Strategy and Action Plan for Mental Health [27] for increasing the number of treated cases in outpatient services. However, the rate of psychiatric beds has decreased from 0.98 to 0.78 per 100,000 inhabitants due to the increase of the population in the period.

There are no community residential facilities for psychiatric patients in the country. This is included in one of the strategies of the Action Plan for Mental Health [27] but is not assumed as a responsibility of the Ministry of Health to provide it. Its responsibility is to promote community and civil society initiatives towards residential support for patients.

There are two Psychiatric hospitals in the country. In 2010 it comprised 173 beds (0.8:100,000), treating around 6.0 patients per 100,000 inhabitants (Table 1). Data from 2014 showed an increase to 298 (1.15 per 100,000 inhabitants).

The main psychiatric hospital located in the capital has capacity for about 400 beds. In 2010 only 120 of these beds were in use due to infrastructure problems in that hospital. Since 2012, in spite of several infrastructural problems persist, the number of beds available has been increasing and in 2014 it reached the 245 beds due to the need for short-term inpatient care.

Since 2010 up to 2014 there are no specific beds for forensic psychiatry; involuntary admissions and isolation or seclusion of patients are common practices but are still not recorded properly in these hospitals.

Most psychiatric hospitals beds were located in the community-based inpatient services but with the increase of the main mental hospital capacity numbers are almost even (298 beds in mental hospitals and 203 in general hospitals). However, most patients are still being treated in outpatient services. Thus, Inpatient care has been delivered on a small scale in psychiatric hospitals and community-based inpatient services.

The female population treated in mental health services represented in 2010 about 40 % of admissions in community-based inpatient services and in outpatient units and there were recorded 29 % of cases in mental hospitals. Children and adolescents are treated in day centers and outpatient units representing respectively 78 and 40 % of the cases in these settings.

Data from WHO-AIMS show that schizophrenia ranks first in admissions either in psychiatric hospitals or in community-based admissions followed by substance abuse and epilepsy. In outpatient services epilepsy and mental disorders due to organic causes are the main causes of patient visits representing 78 % of cases followed by schizophrenia (9 %), neurotic disorders (6 %) and Substance abuse (3 %). (Table 2).

**Table 2:**
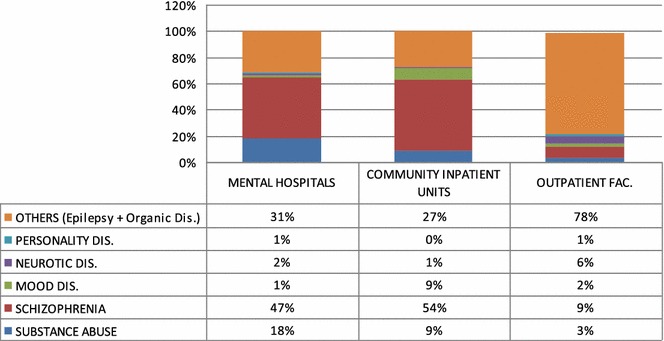
The percentage of patients seen in mental hospitals, community units and outpatient facilities in 2010 [29]

Reports from 2014 show that for the outpatient visits the scenario did not change much in terms of pathologies but there are differences in numbers as epilepsy represents 55.7 % of cases followed by Schizophrenia (19.3 %) and Substance abuse (6.9 %).

In 2010 psychiatric hospitals were the only mental health services where at least one psychotropic drug for every therapeutic group was available. All the psychiatric wards and hospital beds with psychiatric hospitalization experienced breaking stock of psychotropic drugs, especially tranquilizers, anti-depressants and mood stabilizers.

Regarding the outpatient services, over 90 % had no stock of all psychotropic groups throughout the year. This is an issue that was not solved up to 2014. Unfortunately, there have been reported ruptures in medicines stocks including the main psychiatric hospital.

### Human resources

The total number of human resources working in mental health services in 2010 per 100,000 inhabitants was 0.90. According to each professional category, this proportion corresponds to 9 psychiatrists, 2 doctors (not specialized in psychiatry), 44 nurses, 56 psychologists, 5 social workers, 2 occupational therapists and 66 psychiatric technicians (Table 1). Most of the psychiatrists (56 %) work only for the Government (National Health Service) and the remaining (44 %) work for both the State and private institutions. Psychiatrists working stations are mental hospitals and the psychiatric wards in general hospitals.

There were a total of 66 psychiatric technicians in 2010. All of them worked in outpatient units and 12 were also in community-based inpatient units and other 7 were also in psychiatric hospitals. They are not allowed to work in private practice. The mental health coverage to the district level, which is one of the goals of the Strategy and Action Plan for Mental Health, is due to these professionals. Most of outpatient mental health services are provided solely by the psychiatric technicians.

With regard to psychologists, social workers, nurses and occupational therapists, 89 % are linked only to the Government. 6 % of professionals in these areas work both in Government and private institutions. Other 5 % works only in private institutions. The regulation of private psychology practice is weak so data presented are estimates based on records made by the Department of Mental Health team of the Ministry of Health. There are 26 nurses in psychiatric hospitals and 18 in the psychiatric wards in community-based inpatient units. For other mental health professionals, there are 53 psychologists and two occupational therapists in outpatient units. The wards and community-based admissions have 10 psychologists. Psychiatric hospitals have 3 psychologists, 2 social workers and 2 occupational therapists.

In terms of staffing in mental health services, there are 0.02 psychiatrists per bed in the community-based inpatient units and equal proportion in psychiatric hospitals. With regard to nurses, there are 0.09 in the community-based inpatient units and 0.15 in psychiatric hospitals. Psychologists, social workers and occupational therapists are in a ratio of 0.06 by professionals per bed in the community-based inpatient units and 0.04 in psychiatric hospitals. As for psychiatric technicians, the ratio is 0.31 in the community-based inpatient units, and 0.03 in psychiatric hospitals.

Recent data from Ministry of health [26] shows that there are 382 mental health professionals: 9 psychiatrists, 109 psychologists, 241 psychiatric technicians and 23 occupational therapists. This represents an increase of human resources from 0.9 per 100.000 inhabitants in 2010 to 1.48 in 2014. These features allowed the mental health network to expand from 60 to 135 Districts that represents an increase of coverage from 44 to 100 % of the country districts. From 92 health facilities with mental health services, Mozambique has now 153 health facilities including the two psychiatric hospitals and 09 day care centers (Table 1).

### Mental health in primary care: scaling-up mental health services

The training program of health professionals who work in primary care contains less than 2 % of mental health content and the interaction between these professionals and mental health services occurs in less than 20 % of cases as reported in 2010 WHO-AIMS.

Primary health care facilities with or without a physician do not have protocols for evaluation and treatment of mental disorders.

General health care providers receive basic training in mental health during theirs courses and they have the needed knowledge to identify and refer appropriately the mental health cases. General practitioners in particular are also allowed to prescribe a set of psychotropic medicines according to the National Medicines Guidelines [25].

The 2010 report showed that less than 20 % of general practitioners referred patients to mental health services. Currently, due to the expansion of mental health services for all the districts, general health care providers are referring patients to the psychiatric technicians regularly acknowledging their ability to treat mental health patients. It confirms and reassures the important role that psychiatric technicians are playing as part of the general health care provider’s teams [26].

Due to the difficulties in integrating mental health into primary health care services, the National Mental Health Program at Ministry of health decided to train health professionals, named as Psychiatric Technicians, to perform the psychiatric role in primary care level.

These professionals are allowed to prescribe psychotropic drugs of all therapeutic categories and are the mental health professionals who cover mental health in the primary care units in the presence or absence of a physician. Their work includes community interventions, outreach visits, promotion and prevention activities, treatment and follow-up of prisoners, psychosocial counseling and support, home visits and liaison with other health services such as HIV, maternal and child health and non-communicable diseases (diabetes, cardiovascular disorders and oncology). They also interact frequently with traditional healers responding to the country’s health policies considering that people usually seek Traditional Healers’ advice as first contact.

Psychiatric technicians have been trained in the country since 1996. At that time, only the provinces capitals had mental health services [29, 30]. In 2009 coverage expanded to 38 % of the country districts and in 2013 it was 72.6 %. Thanks to the continuous training and supervision of these professionals, the mental health system coverage is now 100 % of the country districts reducing the need of referrals from a province to another and from provinces to mental hospitals located in the extreme north and extreme south of the country.

The achievement of this major goal of the National Program for Mental Health is responding to a primary need of reducing the treatment gap of mental disorders in Mozambican population. The number of patients assisted increased from 29.790 to 97.674 from 2010 to 2014.

However, medicines availability did not grow as the services coverage. Primary care health facilities do not have available throughout the year at least one psychotropic medicine of each therapeutic class (anti-psychotic, anti-depressants, mood stabilizers, anti-epileptics and anxiolytics). A recent study made in Sofala province, centre of Mozambique confirmed that essential psychotropic medicines are routinely unavailable at public health facilities [31]. This is a new challenge for the mental health in Mozambique that needs a prompt innovative solution.

### Monitoring and research

Mental health services regularly collect and compile data on patients treated and services delivered. The Mental Health Department prepares quarterly, half-yearly and annual reports based on data collected at health facilities. There is no research report on mental health held in the country and published in indexed journals in the last 5 years. However, some epidemiological studies have been conducted using clinical and community samples [32].

### Policies and legislation

The WHO-AIMS results from 2010 showed that Mozambique has a strategy and action plan for mental health and its actions have been shaped in the National Health Policy [28, 33]. However, the country does not have a mental health law. Funding for mental health is 0.5 % of the health budget and is basically oriented for treatment services. It is one of the lowest when compared to other health programs such as HIV, internal medicine and circumcision programs that are 16.5, 6.2 and 4.5 % of the health budget respectively [34]. There are no social security plans but the access to health and mental health services is universal and almost free of charge in the National Health Service. Psychotropic medicines prescribed in the public health services are dispensed free of charge; medications availability is sporadic.

The 2010 report stated that the Mental Health Department has the responsibility for the human rights watch. National Mental Health Program is the review body that makes regularly inspections at mental health services, review involuntary admissions, discharges, complaints and investigation processes. Mental heath professionals were trained in mental health and human rights issues in order to address and prevent this type of violations within the health services.

However, there is no independent body or institution extern to the government that makes inspections on human rights on mental health.

In 2014, there are still human rights issues including seclusion, unnecessary long stay inpatient and compulsory inpatient. The number of inpatient beds is very low and the conditions of the hospitals are no adequate. No mental law was approved since then although there were recommendations on this direction. The Ministry of Health is concerned about the human rights of mental health patients and as official institution responsible for the human rights watch in the country; it has developed a mental health law that includes human rights issues and prevention of its violations. However, this law is yet to be approved by the government.

Reports from the Ministry of Health published in 2015 show that the situation related to policies and legislation has not changed in general except for a law for controlling consumption and trade of alcoholic drinks that was approved in 2014. A mental health law is yet to be approved in spite of all advocacies that have been done. Country policies (strategic plan and National Health Policy) are lined up with international legislation on mental health, but there is not yet a law in the country that protects human rights of persons with mental disorders. The mental health system is currently community-based where all the efforts are concentrated in guaranteeing services delivery close to patients houses as recommended by the WHO World Health Report of 2001 [28, 35, 36].

## Discussion

One of the main goals of the Strategy and Action Plan for Mental Health is the expansion of mental health services for all the country districts and reduce treatment gap. This goal was accomplished due to the task-shifting strategy focused on services delivered in primary care by mid-level professionals: the psychiatric technicians.

The implementation of the Psychiatric Technicians from 2010 and 2014 represented an increase of human resources from 0.9 per 100,000 inhabitants to 1.48 in 2014. This increase allowed the mental health network to expand from 60 to 135 Districts, meaning an increase of coverage from 44 to 100 % of the country districts. From 92 health facilities with mental health services, Mozambique has now 153 health facilities including the two psychiatric hospitals and 9 day care centers.

With this increase of human resources, the integration of mental health in other health platforms such as non-communicable diseases, HIV and Maternal and child health became a new goal and challenge. The integration of mental health in NCD is mainly implemented by clinical psychologists in cardiology, oncology, diabetes and pain therapy services while for most cases the psychiatric component is made by psychiatric technicians as liaison psychiatry. Similarly, all mental health professionals are trained to deliver specialized psychosocial interventions for HIV patients at all levels of care. Psychiatric technicians deliver psychosocial support at primary care level for co-morbidity cases in first place but covering all the counseling interventions from HIV pre-test to Anti-retroviral Treatment (ART) management. They are trained to implement basic psychosocial treatments using approaches based on cognitive behavioral therapy (CBT), interpersonal therapy (IPT) and problem solving therapy. With psychiatric technicians and clinical psychologists integrated in the country HIV Program, the psychosocial support for these patients was strengthened due to the inclusion of MH components in the package of care for HIV patients. For the integration of MH in HIV treatment and care program, psychiatric technicians deliver effective psychosocial treatments based on CBT, IPT and Problem solving strategies. These interventions have proven to be effective for depression, to address problems or goals associated with health related behavior change, such as adherence to ART in HIV patients in other settings when delivered by non-specialized health care workers [37].

The main limitation of this study is related to the main source of the data analyzed: the routine data from health facilities. The current information system is not specific for mental health and it allows errors or incomplete data recordings. The presence of Psychiatric Technicians has not always been followed by readily available psychotropic drugs for treatments. The few published articles on Mozambique mental health were published locally and are related to policy, legislation and mental health services organization.

An analysis of the mental health situation in Mozambique shows that the achieved advances have allowed significantly increase of patients seen at the primary care level confirming the usefulness of task-shifting strategy where psychiatrist functions are assigned to mid-level health professionals. The training of mid-level mental health professionals, the psychiatric technicians, allowing them to prescribe psychiatric drugs was the solution found to meet the demand for psychiatric patients at community level.

Integration of mental health into other health care platforms is a new challenge and interventions are delivered mainly by psychiatric technicians at primary health care.

Quality of services delivered is still a challenge. It is a very important part of task-shifting strategy and the specialists and funding available are not enough to guarantee regular supervision and technical support for the psychiatric technicians in primary care.

## Conclusions

In Mozambique, as happens in most LAMICs, the treatment gap of mental disorders is due in part to lack of qualified human resources. The task-shifting approach has become an alternative solution that has been proven effective in reducing this gap.

The training of psychiatric technicians in Mozambique that began in 2006, at a time when there was not yet enough evidence on its effectiveness, was the solution adopted by the Ministry of Health, in a country that had only one national psychiatrist.

Currently, mental health services have multidisciplinary teams of psychiatrists (9), psychologists (109), occupational therapists (23) and psychiatric technicians (241) for 25.727.911 inhabitants. However, only the main hospitals have all categories of professionals simultaneously. In primary care, task-shifting allowed psychiatric technicians to delivery all mental health care, including prescription of psychotropic drugs.

The 241 psychiatric technicians in the country today have increased the coverage of mental health services from 60 (44 %) districts in 2010 to 135 (100 %) in 2015. As a result, the number of patients seen has also increased from 29.790 in the year 2010 to 97.674 people in 2010 and thus reducing the treatment gap in terms of access to mental health services.
